# Supplementary material for: Evolution and structure of clinically relevant gene fusions in multiple myeloma
Source: Nat Commun. 2020 May 29;11:2666. doi: 10.1038/s41467-020-16434-y (PMC7260243; doi:10.1038/s41467-020-16434-y)
Supplement: Supplementary file 1 — Supplementary Information [file 41467_2020_16434_MOESM1_ESM.pdf]

Evolution and structure of clinically relevant gene fusions in multiple myeloma
Foltz et al.

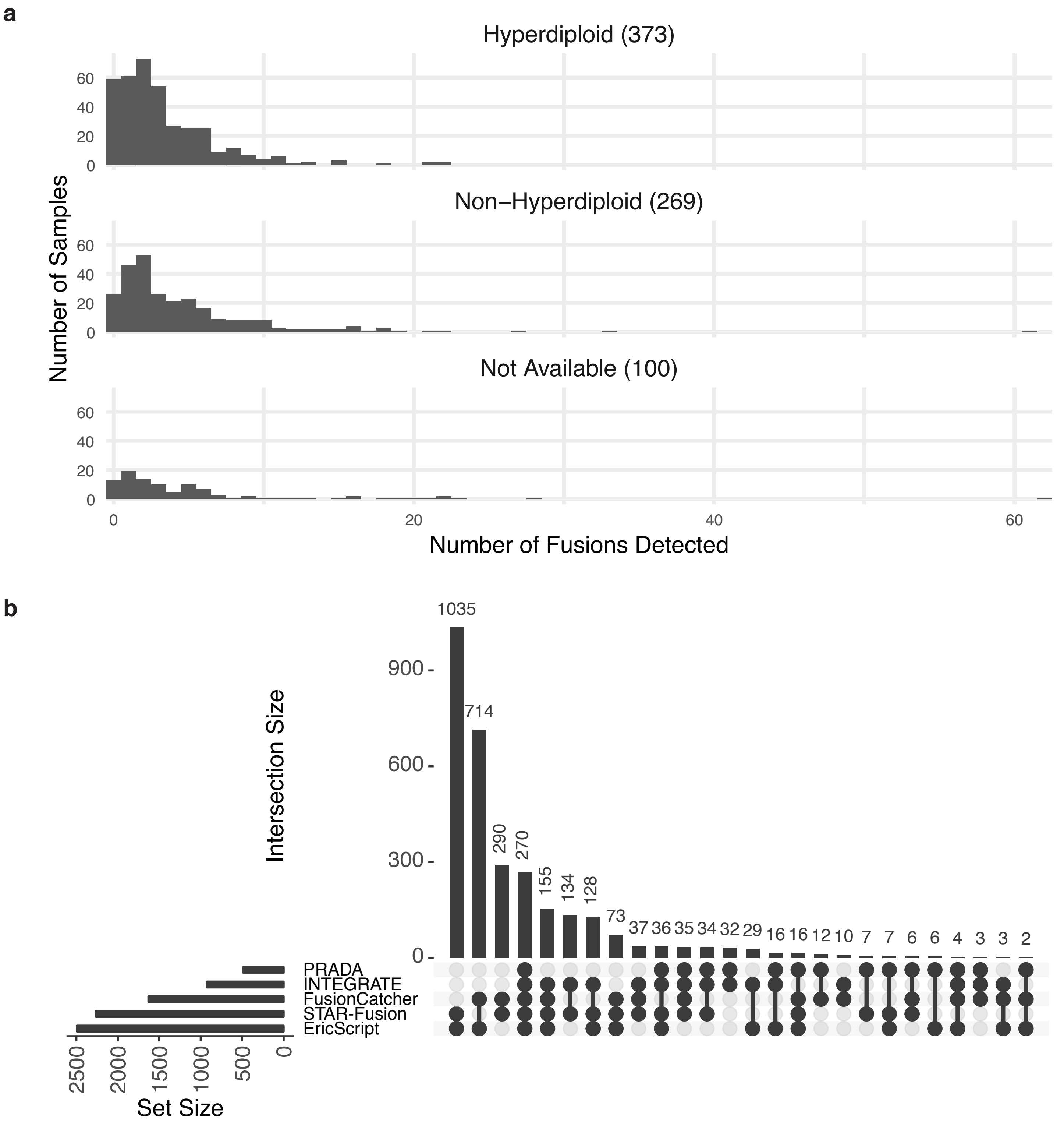

Supplementary Figure 1. Related to Figure 1. a. Number of fusions detected per sample, stratified by hyperdiploid status. **b.** Fusion caller overlap after filtering. Source data and scripts are available at doi.org/10.6084/m9.figshare.11941494.

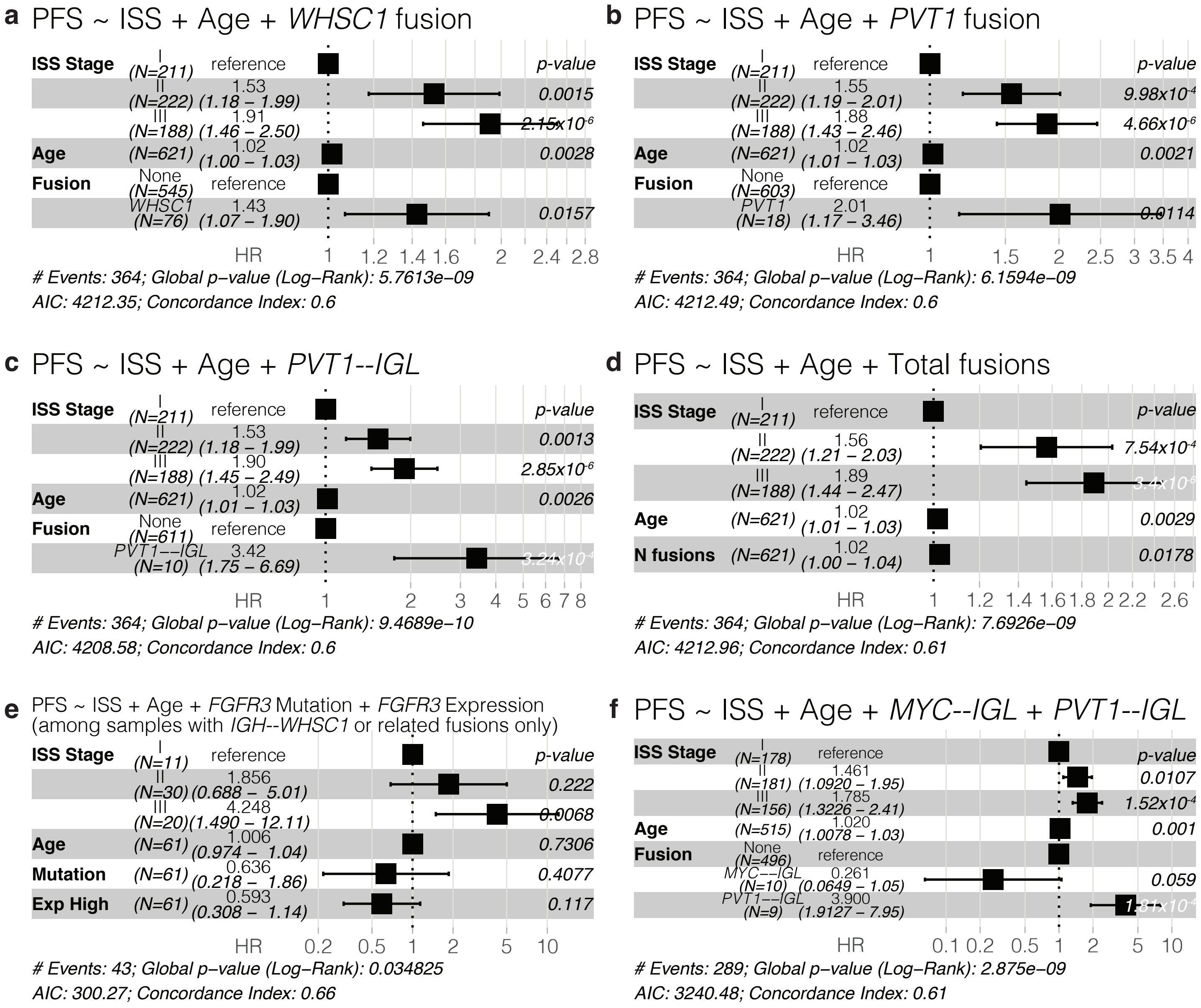

Supplementary Figure 2. Related to Figures 1, 5, 6. a-f. Progression-free survival (PFS) models using multivariate Cox proportional hazards. Error bars indicate a 95% confidence interval on each hazard ratio estimate. Covariate p-values derived from z-scores are two-sided. Source data and scripts are available at doi.org/10.6084/m9.figshare.11941494.

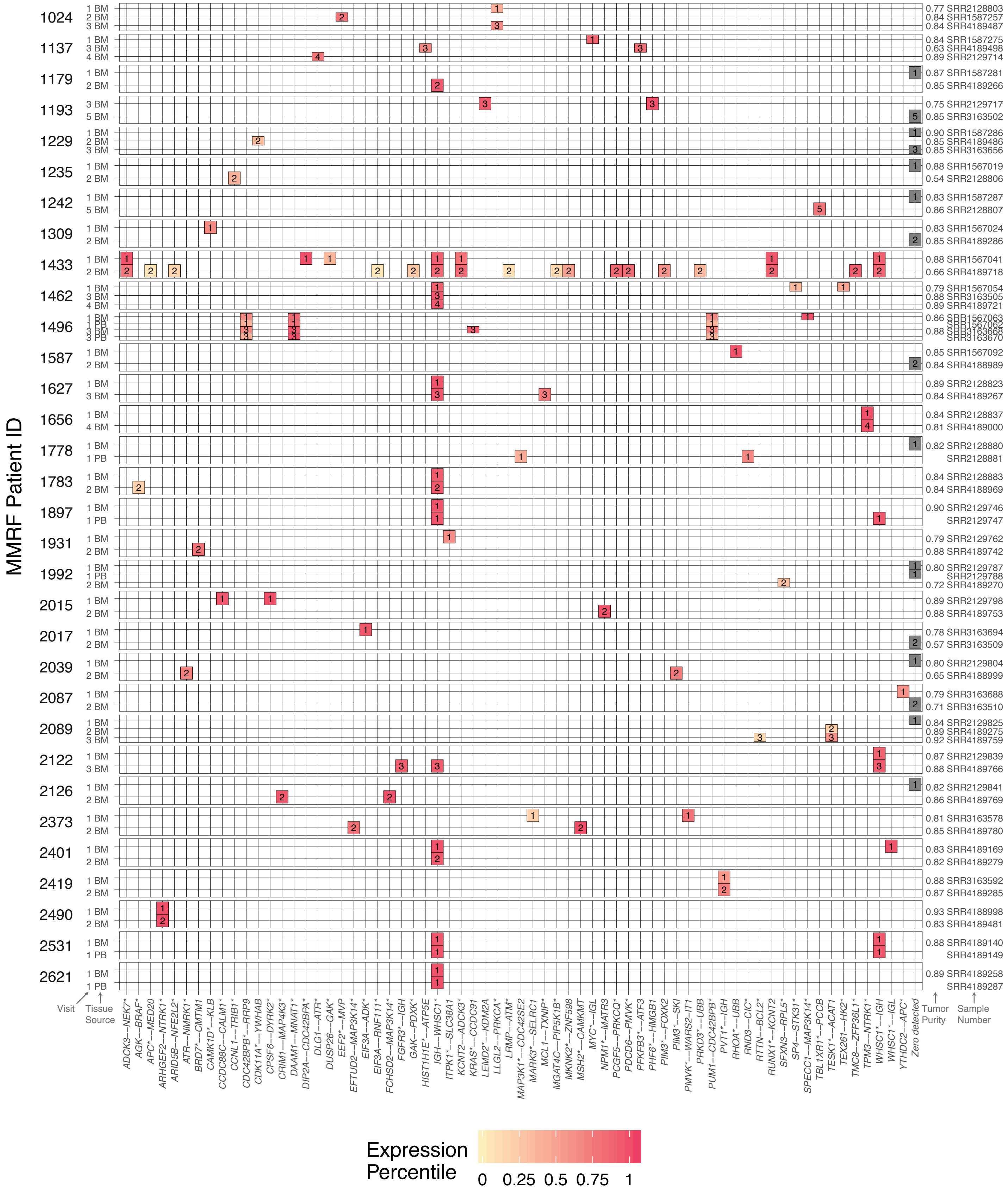

Supplementary Figure 3. Related to Figure 3. Expression percentile of cancer-related genes (marked with *) in patients with samples from multiple clinic visits or tissue sites (bone marrow, BM, or peripheral blood, PB). Tumor purity of PB samples was not quantified. Source data and scripts are available at doi.org/10.6084/m9.figshare.11941494.

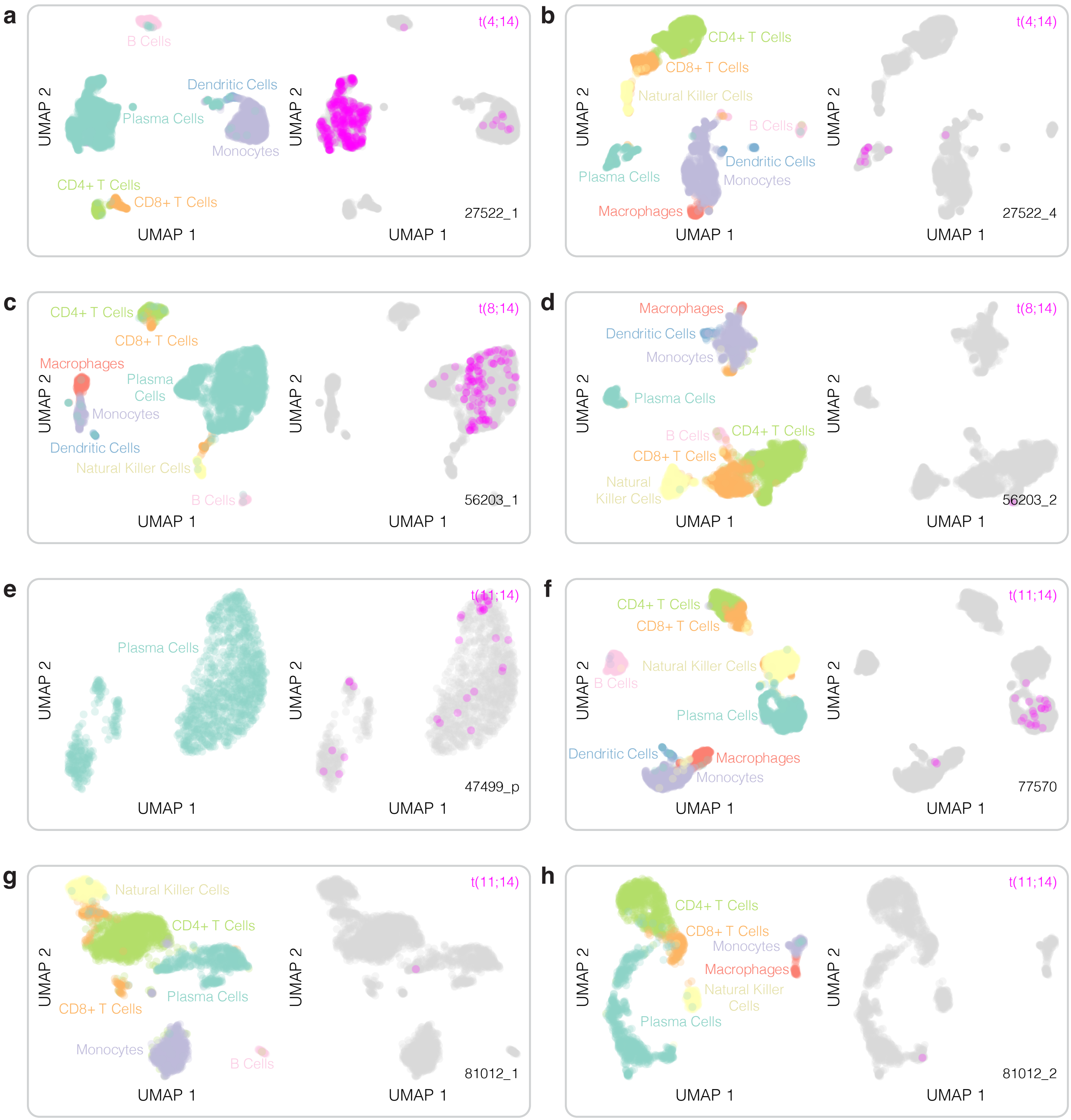

Supplementary Figure 4. Related to Figure 4. a-b. Cell types and Fuscia scRNA-seq fusion discovery for Patient 27522 (primary, relapse) with t(4;14). Results from overlapping and non-overlapping regions. **c-d.** Patient 56203 (primary, relapse) with t(8;14). **e.** Patient 47499 (CD138+ sorted primary) with t(11;14). **f.** Patient 77570 (primary) with t(11;14). **g-h.** Patient 81012 (primary, relapse) with t(11;14). Source data and scripts are available at doi.org/10.6084/m9.figshare.11941494 and doi.org/10.6084/m9.figshare.11941506.

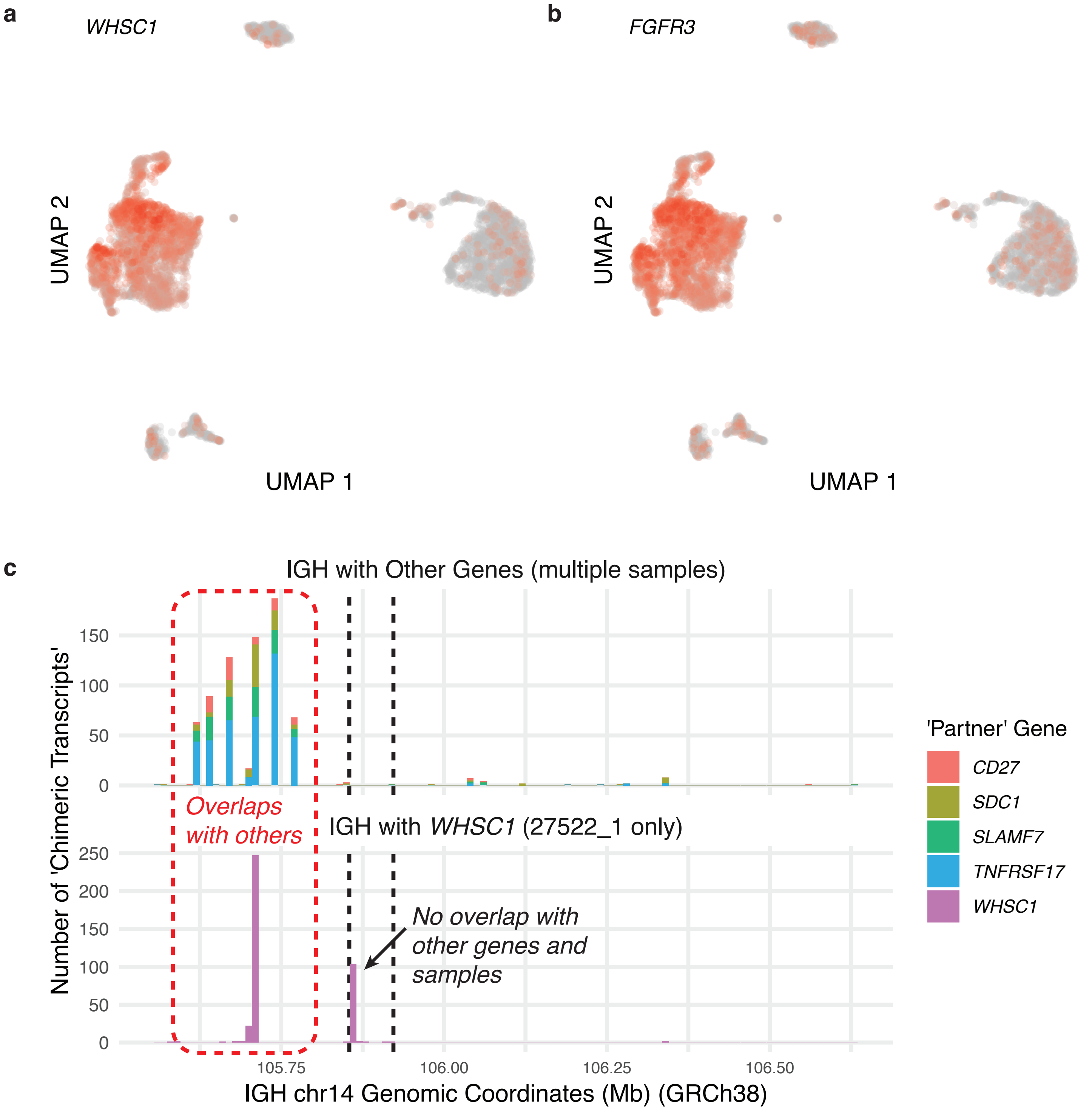

Supplementary Figure 5. Related to Figure 4. a. Single cell expression of *WHSC1*. **b.** Single cell expression of *FGFR3*. **c.** Mapping location and number of 'Chimeric Transcripts' linking IGH with various 'Partner' genes which do not form real fusions with IGH except for *WHSC1*. Source data and scripts are available at doi.org/10.6084/m9.figshare.11941494.

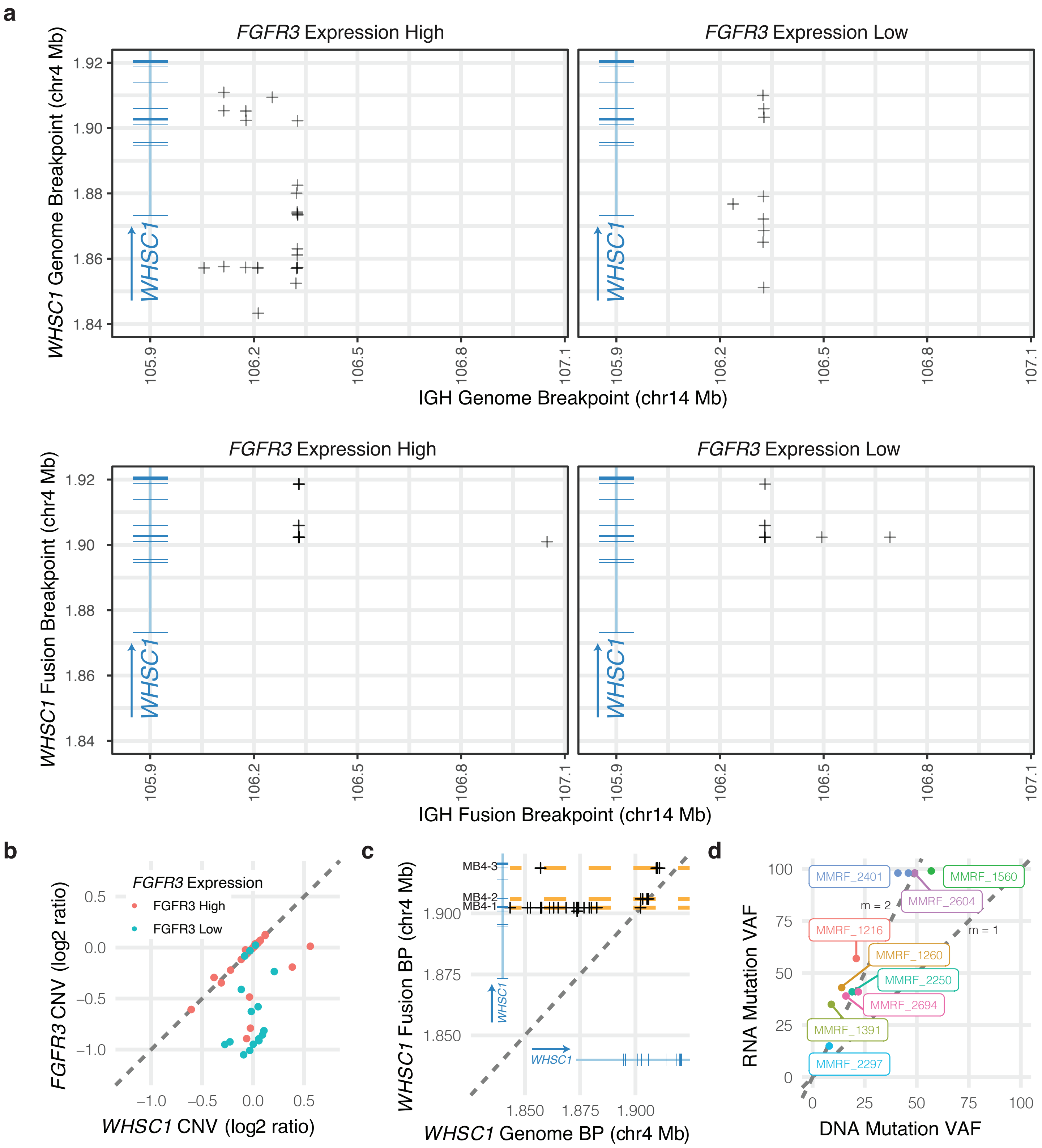

Supplementary Figure 6. Related to Figure 5. a. Breakpoint mapping locations at IGH and *WHSC1*, split by *FGFR3* expression. **b.** *WHSC1* and *FGFR3* copy number in samples with IGH fusion. **c.** Breakpoint (BP) mapping location at *WHSC1*. **d.** *FGFR3* DNA and RNA variant allele frequency (VAF) comparison. Source data and scripts are available at doi.org/10.6084/m9.figshare.11941494.

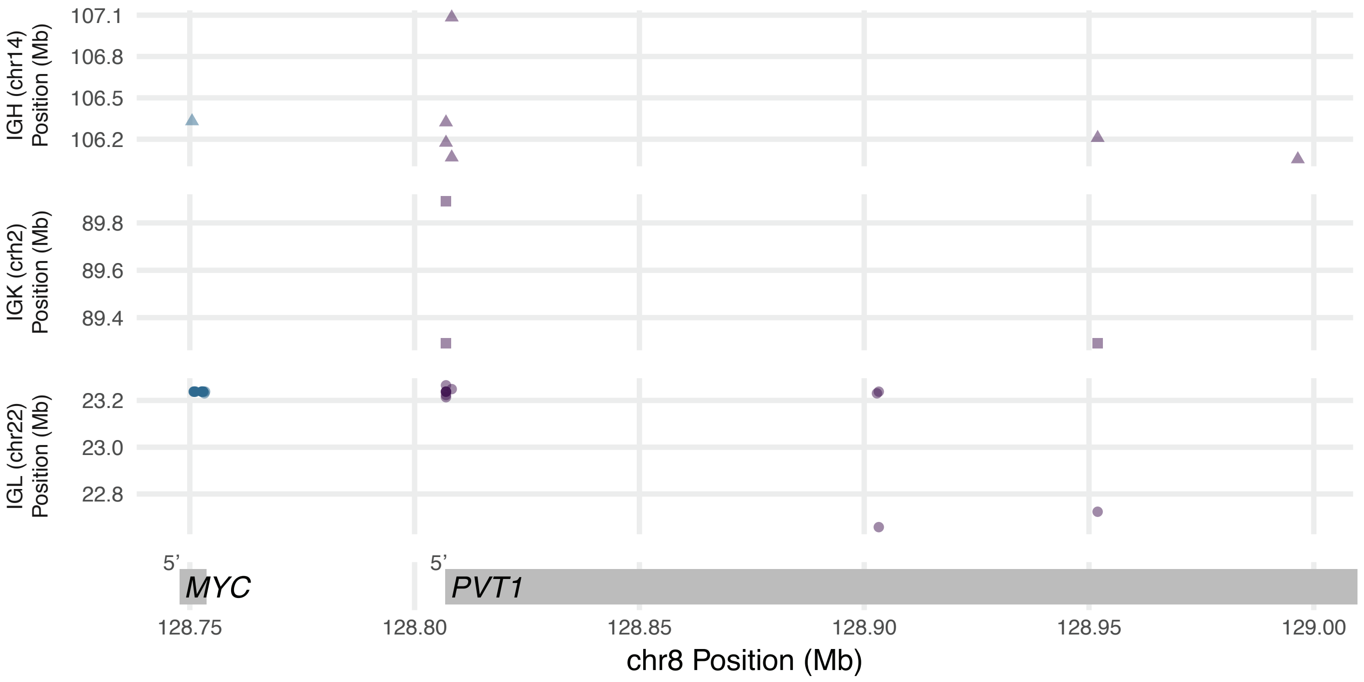

Supplementary Figure 7. Related to Figure 6. Fusion breakpoint mapping locations at Ig loci, *MYC*, and *PVT1*. Source data and scripts are available at doi.org/10.6084/m9.figshare.11941494.

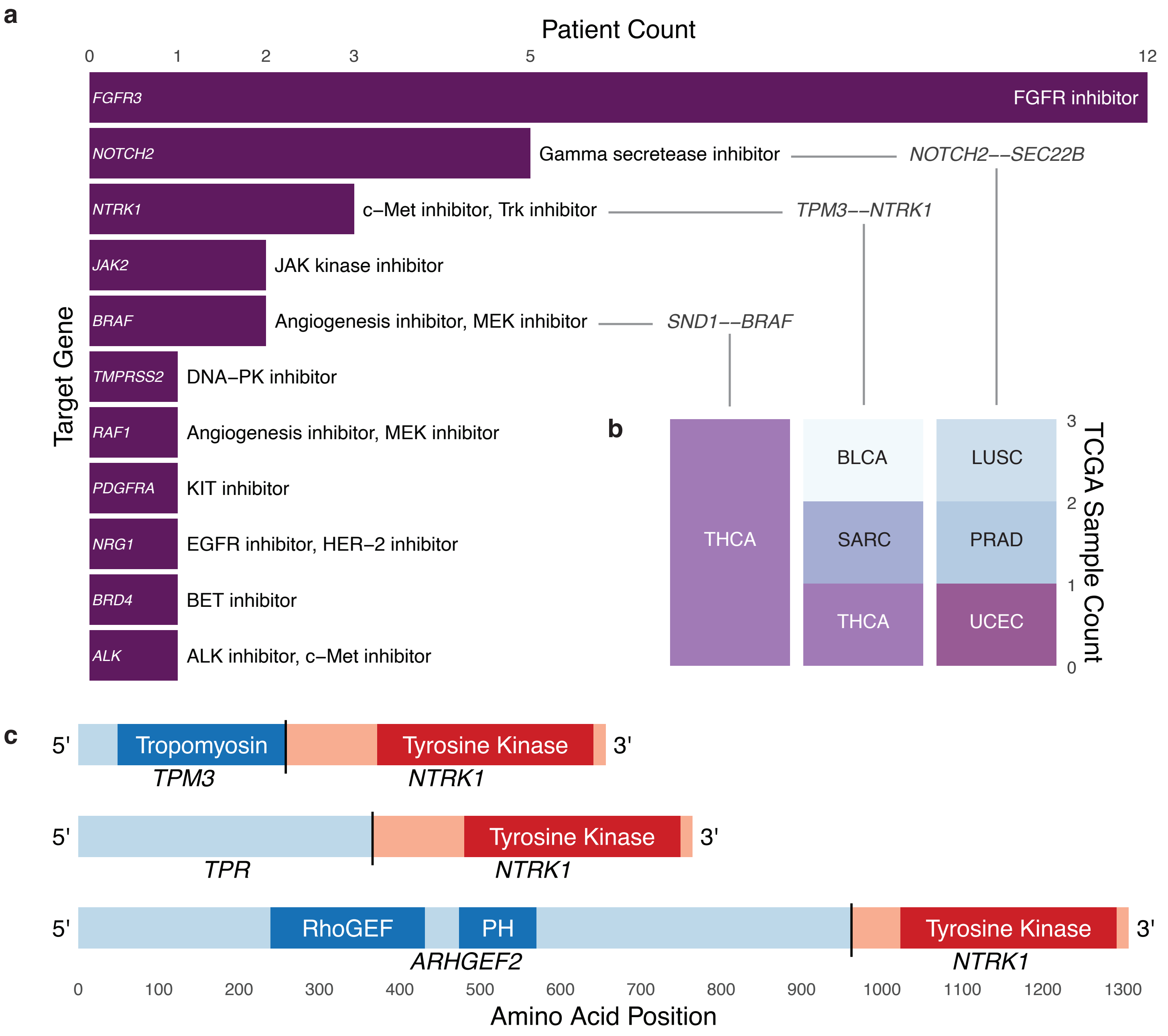

Supplementary Figure 8. a. Overlap of fusion calls with DEPO drug target database. **b.** Cancer types with exact fusion overlaps. **c.** Protein structures of *NTRK1* gene fusions. Source data and scripts are available at doi.org/10.6084/m9.figshare.11941494.

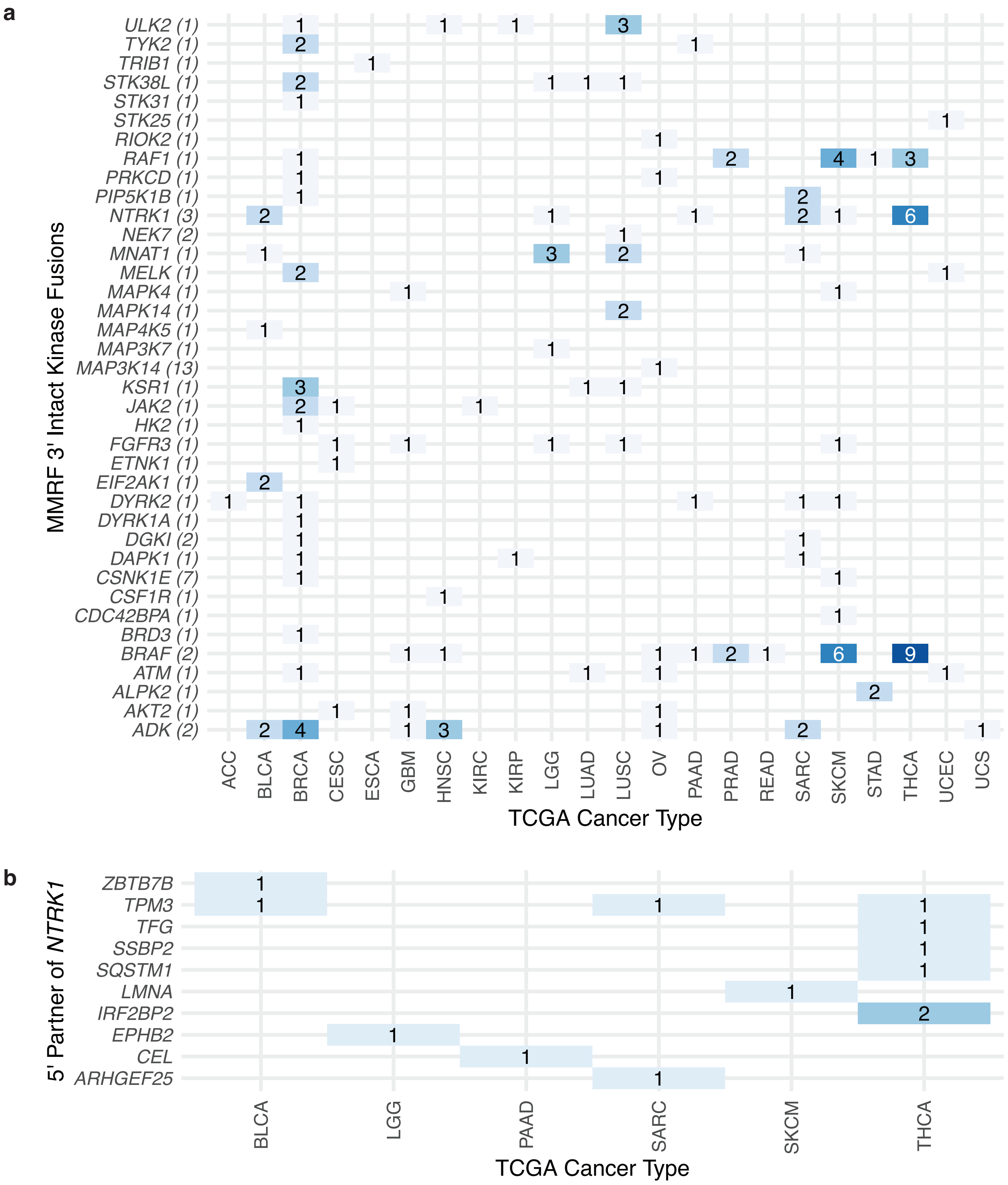

Supplementary Figure 9. a. MMRF 3' intact kinase fusion partner genes overlapping with TCGA cancer types (number of samples). **b.** MMRF *NTRK1* partner genes overlapping with TCGA cancer types. Source data and scripts are available at doi.org/10.6084/m9.figshare.11941494.
