# Supplementary material for: Evolution and structure of clinically relevant gene fusions in multiple myeloma
Source: Nat Commun. 2020 May 29;11:2666. doi: 10.1038/s41467-020-16434-y (PMC7260243; doi:10.1038/s41467-020-16434-y)
Supplement: Supplementary file 3 — Description of Additional Supplementary Files [file 41467_2020_16434_MOESM3_ESM.pdf]

Description of Additional Supplementary Files

File Name: Supplementary Data 1
Description: **Patient Info.** Patient clinical information and sequencing data availability.

File Name: Supplementary Data 2
Description: **Patient list.** List of all patients and samples included.

File Name: Supplementary Data 3
Description: **Fusion Calls.** Fusion calls from primary and secondary samples.

File Name: Supplementary Data 4
Description: **Overexpressed.** Genes showing significant overexpression in fusions samples. Association testing was done using Student's t-test (two-sided) (continuous expression) and Fisher's Exact Test (two-sided) (categorical expression).

File Name: Supplementary Data 5
Description: **Kinases.** Kinase fusion annotation including intact kinase domains.

File Name: Supplementary Data 6
Description: **Fusion Filtering.** Fusions removed from analysis through soft filters.
