# Supplementary material for: Evolution and structure of clinically relevant gene fusions in multiple myeloma
Source: Nat Commun. 2020 May 29;11:2666. doi: 10.1038/s41467-020-16434-y (PMC7260243; doi:10.1038/s41467-020-16434-y)
Supplement: Supplementary file 10 — Reporting Summary [file 41467_2020_16434_MOESM10_ESM.pdf]

nature research

Corresponding author(s): Li Ding, Ravi Vij

Last updated by author(s): Mar 5, 2020

Reporting Summary

Nature Research wishes to improve the reproducibility of the work that we publish. This form provides structure for consistency and transparency in reporting. For further information on Nature Research policies, see Authors & Referees and the Editorial Policy Checklist .

Statistics

For all statistical analyses, confirm that the following items are present in the figure legend, table legend, main text, or Methods section.

n/a	Confirmed	
☐	☒	The exact sample size (*n*) for each experimental group/condition, given as a discrete number and unit of measurement
☐	☒	A statement on whether measurements were taken from distinct samples or whether the same sample was measured repeatedly
☐	☒	The statistical test(s) used AND whether they are one- or two-sided<br>*Only common tests should be described solely by name; describe more complex techniques in the Methods section.*
☐	☒	A description of all covariates tested
☐	☒	A description of any assumptions or corrections, such as tests of normality and adjustment for multiple comparisons
☐	☒	A full description of the statistical parameters including central tendency (e.g. means) or other basic estimates (e.g. regression coefficient) AND variation (e.g. standard deviation) or associated estimates of uncertainty (e.g. confidence intervals)
☐	☒	For null hypothesis testing, the test statistic (e.g. *F*, *t*, *r*) with confidence intervals, effect sizes, degrees of freedom and *P* value noted<br>*Give P values as exact values whenever suitable.*
☒	☐	For Bayesian analysis, information on the choice of priors and Markov chain Monte Carlo settings
☒	☐	For hierarchical and complex designs, identification of the appropriate level for tests and full reporting of outcomes
☐	☒	Estimates of effect sizes (e.g. Cohen's *d*, Pearson's *r*), indicating how they were calculated

Our web collection on statistics for biologists contains articles on many of the points above.

Software and code

Policy information about availability of computer code

Data collection

Alignment
Paired RNA-seq fastq files were aligned to GRCh37 using STAR version 2.5.3a_modified.
BAM files were sorted and analyzed with flagstat using Samtools version 1.5.
Quality control was conducted using FastQC version 0.11.5. (See bioinformatics.babraham.ac.uk/projects/fastqc/.)

Association testing and correlation
Association testing was done using Student's t-test (continuous expression) and Fisher's Exact Test (categorical expression). Clinical associations with fusions and fusion genes were calculated using Fisher's Exact Test for categorical variables and Mann-Whitney U Test for continuous variables. Expression and clinical testing p-values were corrected using the Benjamini and Hochberg false discovery rate (FDR) method. All correlations are calculated as Pearson correlations unless otherwise stated.

Copy number variation detection
We detected copy number variation from WGS data using BIC-seq2 (BICseq2-norm version 0.2.4; BICseq2-seg version 0.7.2). In scRNA-seq, we used inferCNV (version 0.8.2) to calculate single cell copy number profiles.

Fusion analysis scripts
Fusion results were analyzed by scripts written in Python (version 3.7.2) and R (version 3.5.3). Python packages included numpy, os, and pysam. R packages included ggrepel, gridExtra, readxl, RColorBrewer, Seurat (version 3.0.0), survival, survminer, tidyverse, and UpSetR. (Please see github.com/ding-lab/griffin-fusion/tree/master/mmrf_fusion for fusion analysis scripts.)

Fusion detection
We used five fusion detection tools including EricScript (version 0.5.5), FusionCatcher (version 1.00), INTEGRATE (version 0.2.6, using RNA-seq samples only, not paired RNA and WGS), PRADA (version 1.2), and STAR-Fusion (version 1.1.0). Gene names from immunoglobulin super-loci were condensed to IGH, IGK, and IGL (including IGLL5).

Fusion filtering
Fusions were required to be called by at least two tools. Fusions called by any combination of EricScript, FusionCatcher, or INTEGRATE must also have been called by STAR-Fusion or PRADA in another sample (soft filter tag EFI). Fusions were removed if: partners are the same gene; genes appear on blacklist or are paralogs; fusion comes from list of normal panel fusions (non-cancer cell lines, GTEx, TCGA normal samples); one partner is promiscuous with 25 or more partners (soft filter tag Many Partners); or partner genes are within 300 Kb (soft filter tag Within 300Kb). Additionally, across all samples for a particular fusion pair, we required at least one sample to have 2 or more junction reads or one sample to have 1 or more spanning reads, or that fusion pair was removed from all samples (soft filter tag Low Count). Finally, fusions with a low WGS support rate compared to the background rate were removed if the binomial test p-value was less than 0.15 (soft filter tag Undervalidated). See Supplementary Table 6 for a list of all “soft filtered” fusions and why they were filtered.

Gene expression
Transcripts per million (TPM) was calculated using kallisto (version 0.43.1).
Gene level TPM was calculated as the sum of TPM values from each of that gene’s transcripts.
Log transformation of TPM values was calculated as log10(TPM + 1).

Kinase domain analysis
Kinase domain status was determined based on reported gene fusion breakpoints using AGFusion (version 1.231). (See github.com/murphycj/AGFusion.) Following manual review, 15 out of 19 MAP3K14 fusions were found to possess an intact kinase domain after initially being reported as having disrupted kinase domains due to a lack of annotation.

Mutation signature profiling
We used SignatureAnalyzer to quantify mutation signatures.

Outlier detection
Gene expression outliers were defined as having values greater than 75th + 1.5*IQR or less than 25th - 1.5*IQR, where 75th and 25th represent the 75th and 25th percentile, respectively, and IQR is the interquartile range, defined as the 75th percentile minus the 25th percentile.

Single cell fusion detection -- Fuscia
Given an aligned BAM file, barcode information for each read mapping to fusion gene regions was extracted using the Python module pysam (version 0.15.2), which wraps Samtools (version 1.7). When two reads map to different genes or regions and share the same cell and molecular barcode, we labeled that transcript as a “chimeric transcript”. Multiple reads could originate from the same chimeric transcript. We eliminated reads with length > 128 and then selected one representative read from each side of the chimeric transcript by picking the reads mapping closest to the known WGS breakpoint. Transcript overexpression makes false positive detection of chimeric transcripts more likely. We reduced this risk by purposefully looking for chimeric transcripts that may be detected due to overexpression. In plasma cells with IGH translocations, we specifically looked for chimeric transcripts linking IGH and plasma cell markers SDC1, SLAMF7, and TNFRSF17. We called those regions ‘overlap’ regions because chimeric transcripts from genes not associated with fusions overlap with those from legitimate fusions. (Please see github.com/ding-lab/fuscia.)

We used R (version 3.5.3) and the Seurat package (version 3.0.0) to analyze cell type and gene expression from individual data. Dimensional reduction was performed using UMAP.

Somatic mutation calling
MMRF exome bams were aligned to hg19, and somatic variants were called by our in-house pipeline SomaticWrapper, which includes four established bioinformatic tools (Mutect (version 1.1.7), Pindel (version 0.2.54), Strelka2 (version 2.9.2), and VarScan2 (version 2.3.83)). (See github.com/ding-lab/somaticwrapper.) We kept SNVs called by at least two out of three tools (Mutect, Strelka, VarScan2). Likewise, we kept INDELs called by at least two out of three tools (Pindel, Strelka, VarScan2). We required 14X coverage for somatic mutation calls and only kept mutations with tumor variant allele frequency (VAF) >= 0.05 and normal VAF <= 0.02.

Structural variant detection
Structural variants were detected from paired normal and tumor WGS samples using Delly (version 0.7.6) and Manta (version 1.1.0). To be analyzed, tumor and normal WGS samples must have had matching sequencing assays and a corresponding RNA-seq sample.

Survival analysis
We performed survival analysis using progression-free survival as the outcome using the survival (version 2.44-1.1) and survminer (version 0.4.6) packages in R. To test for significant improvements in model fit with additional covariates, we implemented a chi-squared test using the anova() function and compared the new model to the baseline model.

Tumor purity
We used the R package estimate (version 2.0) to quantify tumor purity from RNA-seq data. Tumor purity of peripheral blood (PB) samples was not quantified.

WGS support of fusion events
We used WGS data to determine if reported fusions also had genomic support. We defined a breakpoint window centered at each fusion breakpoint. If there were 3 or more discordant read pairs mapping to within 100 Kb of each breakpoint, we determined the fusion to be supported by WGS. Reads were filtered by Samtools (version 1.5) with flags -F 1920 -f 1 -q 20.

Data analysis

Fusion analysis scripts
Fusion results were analyzed by scripts written in Python (version 3.7.2) and R (version 3.5.3). Python packages included numpy, os, and

pysam. R packages included ggrepel, gridExtra, readxl, RColorBrewer, Seurat (version 3.0.0), survival, survminer, tidyverse, and UpSetR.

Data analysis scripts and single cell fusion detection methods are available under the MIT license at github.com/ding-lab/griffin-fusion/tree/master/mmrf_fusion and github.com/ding-lab/fuscia.

For manuscripts utilizing custom algorithms or software that are central to the research but not yet described in published literature, software must be made available to editors/reviewers. We strongly encourage code deposition in a community repository (e.g. GitHub). See the Nature Research guidelines for submitting code & software for further information.

Data

Policy information about availability of data

All manuscripts must include a data availability statement. This statement should provide the following information, where applicable:

- Accession codes, unique identifiers, or web links for publicly available datasets
- A list of figures that have associated raw data
- A description of any restrictions on data availability

Data was provided by The Multiple Myeloma Research Foundation (MMRF) CoMMpass (Relating Clinical Outcomes in MM to Personal Assessment of Genetic Profile) Study (NCT01454297). dbGaP Study Accession: phs000748.

For single cell RNA sequencing of additional patient samples, the Washington University Institutional Review Board approved the study protocol, and we have complied with all relevant ethical regulations, including obtaining informed consent from all participants.

The source data underlying all figures are provided as Source Data files accessible with DOIs 10.6084/m9.figshare.11941494 (for everything except scRNA data) and 10.6084/m9.figshare.11941506 (for scRNA data).

Field-specific reporting

Please select the one below that is the best fit for your research. If you are not sure, read the appropriate sections before making your selection.

☒ Life sciences ☐ Behavioural & social sciences ☐ Ecological, evolutionary & environmental sciences

For a reference copy of the document with all sections, see nature.com/documents/nr-reporting-summary-flat.pdf

Life sciences study design

All studies must disclose on these points even when the disclosure is negative.

Sample size	Sample size was determined by the availability of RNA-seq data for patients enrolled in the MMRF CoMMpass Study.
Data exclusions	Data from patient MMRF_1108 (RNA-seq sample SRR1567010) was excluded due to repeated failure of fusion detection tools to run on this sample.
Replication	No measures were taken to verify the reproducibility of the experimental findings. Replication measures were not relevant to the study design.
Randomization	Samples were not randomized into experimental groups. Randomization was not relevant to the study design.
Blinding	Investigators were not blinded to group allocation. Blinding was not relevant to the study design.

Reporting for specific materials, systems and methods

We require information from authors about some types of materials, experimental systems and methods used in many studies. Here, indicate whether each material, system or method listed is relevant to your study. If you are not sure if a list item applies to your research, read the appropriate section before selecting a response.

Materials & experimental systems

n/a	Involved in the study	
☒	☐	Antibodies
☒	☐	Eukaryotic cell lines
☒	☐	Palaeontology
☒	☐	Animals and other organisms
☐	☒	Human research participants
☐	☒	Clinical data

Methods

n/a	Involved in the study	
☒	☐	ChIP-seq
☒	☐	Flow cytometry
☒	☐	MRI-based neuroimaging

Human research participants

Policy information about studies involving human research participants

Population characteristics	For single cell RNA sequencing of additional patient samples, patients were diagnosed with multiple myeloma and were included in this analysis based on testing positive for a translocation relevant to single cell fusion detection (t(4;14), t(8;14), or t(11;14)).
Recruitment	Patients were recruited for banking of clinical specimens on a prospective IRB approved protocol.
Ethics oversight	Washington University Institutional Review Board

Note that full information on the approval of the study protocol must also be provided in the manuscript.

Clinical data

Policy information about clinical studies

All manuscripts should comply with the ICMJE guidelines for publication of clinical research and a completed CONSORT checklist must be included with all submissions.

Clinical trial registration	Data was provided by The Multiple Myeloma Research Foundation (MMRF) CoMMpass (Relating Clinical Outcomes in MM to Personal Assessment of Genetic Profile) Study (NCT01454297). dbGaP Study Accession: phs000748.
Study protocol	https://www.ncbi.nlm.nih.gov/projects/gap/cgi-bin/study.cgi?study_id=phs000748.v1.p1
Data collection	*Describe the settings and locales of data collection, noting the time periods of recruitment and data collection.*
Outcomes	*Describe how you pre-defined primary and secondary outcome measures and how you assessed these measures.*
